# Evaluation of the performance of Abbott m2000 and Roche COBAS Ampliprep/COBAS Taqman assays for HIV-1 viral load determination using dried blood spots and dried plasma spots in Kenya

**DOI:** 10.1371/journal.pone.0179316

**Published:** 2017-06-16

**Authors:** Clement Zeh, Kenneth Ndiege, Seth Inzaule, Rebecca Achieng, John Williamson, Joy Chih-Wei Chang, Dennis Ellenberger, John Nkengasong

**Affiliations:** 1 Division of HIV/AIDS Prevention, U.S. Centers for Disease Control and Prevention, Kisumu, Kenya; 2 Centre for Global Health Research, Kenya Medical Research Institute, Kisumu, Kenya; 3 International Laboratory Branch, Division of Global HIV/AIDS, Center for Global Health, U.S. Centers for Disease Control and Prevention, Atlanta, Georgia, United States of America; Public Health Agency of Canada, CANADA

## Abstract

**Background:**

Routine HIV viral load testing is not widely accessible in most resource-limited settings, including Kenya. To increase access to viral load testing, alternative sample types like dried blood spots (DBS), which overcome the logistic barriers associated with plasma separation and cold chain shipment need to be considered and evaluated. The current study evaluated matched dried blood spots (DBS) and dried plasma spots (DPS) against plasma using the Abbott M 2000 (Abbott) and Roche Cobas Ampliprep/Cobas TaqMan (CAP/CTM) quantitative viral load assays in western Kenya.

**Methods:**

Matched plasma DBS and DPS were obtained from 200 HIV-1 infected antiretroviral treatment (ART)-experienced patients attending patient support centers in Western Kenya. Standard quantitative assay performance parameters with accompanying 95% confidence intervals (CI) were assessed at the assays lower detection limit (400cps/ml for CAP/CTM and 550cps/ml for Abbott) using SAS version 9.2. Receiver operating curves (ROC) were further used to assess viral-load thresholds with best assay performance (reference assay CAP/CTM plasma).

**Results:**

Using the Abbott test, the sensitivity and specificity, respectively, for DPS were (97.3%, [95%CI: 93.2–99.2] and 98.1% [95%CI: 89.7–100]) and those for DBS (93.9% [95%CI: 88.8–97.2] and 88.0% [95%CI: 82.2–92.4]). The correlation and agreement using paired plasma and DPS/DBS were strong, with r^2^ = 90.5 and r_c_ = 68.1. The Bland-Altman relative percent change was 95.3 for DPS, (95%CI: 90.4–97.7) and 73.6 (95%CI: 51.6–86.5) for DBS. Using the CAP/CTM assay, the sensitivity for DBS was significantly higher compared to DPS (100.0% [95% CI: 97.6–100.0] vs. 94.7% [95%CI: 89.8–97.7]), while the specificity for DBS was lower: 4%, [95% CI: 0.4–13.7] compared to DPS: 94.0%, [95% CI: 83.5–98.7]. When compared under different clinical relevant thresholds, the accuracy for the Abbott assay was 95% at the 1000cps/ml cut-off with a sensitivity and specificity of 96.6% [95% CI 91.8–98.7] and 90.4% [95% CI 78.2–96.4] respectively. The optimum threshold was at 3000 cps/ml with an accuracy of 95.5%, sensitivity and specificity of 94.6% [95%CI 89.3–97.5] and 98.1% [95%CI 88.4–99.9]) respectively. The best threshold for CAP/CTM was at 4000 copies /mL, with 92.5% accuracy (sensitivity of 96.0% [95%CI 91.0–98.3] and specificity of 82.7% [95%CI 69.2–91.3]).

**Conclusions:**

There was similar performance between matched DBS, DPS and plasma using the Abbott test, and good correlation for matched DPS and plasma using the CAPCTM test. The findings suggest that DBS and DPS may be reliably used as alternative specimens to plasma to measure HIV-1 VL using Abbott, and DPS may be reliably used with CAP/CTM in resource-limited settings.

## Background

More than 60% of HIV-1 infected individuals reside in Africa[1]. HIV is the leading cause of death in sub-Saharan Africa and a major cause of mortality globally[1]. Among the various interventions to prevent spread of HIV, consistent use of antiretroviral therapy (ART) has been demonstrated to be the one of the most efficacious, with a 96% reduction in the rate of linked transmission among discordant couples in one study[2]. Data from previous studies have suggested that the infected person’s VL needs to be reduced below 1000–1500 copies /mL for the likelihood of transmission to approach zero[3]. Approximately 9.1 million of infected persons are on antiretroviral therapy in sub-Saharan Africa, and the success of the treatment depends on routine monitoring[4]. Due to limited resources, previous treatment guidelines recommended the use of immunological and clinical markers for treatment monitoring[5]. These markers have however been shown to be poor surrogates leading to misclassification of treatment failure, accumulation of HIV-1 drug resistant strains subsequently limiting future treatment options, or resulting with unwarranted switch to an expensive regimen[6–8].The alternative use of VL for monitoring patients on ART has several advantages including more timely and accurate detection of virologic treatment failure and appropriate adjustments in treatment, which may prevent antiretroviral resistance and secure future treatment options. This may also contribute to a reduction in the overall cost of care[9]. Consequently, WHO guidelines strongly recommend use of routine VL testing for ART treatment failure monitoring where available, including resource-limited settings (RLS)[3]. The Kenyan Ministry of health has adopted this recommendation, which has resulted in programmatic scale-up of VL testing[10].

However there are still challenges limiting access to VL testing by the majority of HIV-1 infected individuals on ART in Kenya. Among the obstacles are stringent costs associated with laboratory infrastructure, shipping of specimens to the few reference laboratories with capacity for VL testing, and frequent delays in receipt of results from such laboratories. The use of plasma, currently used for VL testing in RLS, is hampered by the need of preparation from whole blood specimens, storage requirements, and cold-chain transportation from usually remote areas to the centralized testing facilities. Therefore, there is need for a more feasible approach, such as the use of alternative specimen types, to help reduce costs and increase access to VL testing.

Dried fluid specimens (DFS) including dried blood specimens (DBS) and dried plasma specimens (DPS) can provide several logistical and practical advantages to plasma including easy collection (specifically DBS) transportation, and storage. DFS can also overcome the need for cold-shipment, and have been shown to be stable at room temperature without degradation of the viral nucleic acids[11]. In children, among whom the preferred sample collection is through finger or heel prick, DBS offers the advantage of small volume requirement compared to venipuncture[12]. In addition, previous findings have shown promising results for viral-load testing with DFS, with results comparable to those derived from plasma in VL testing[13–18]. Despite these advantages, DFS have still not been widely adopted into routine VL testing suggesting the need for more data to guide implementation. The objective of this study was to evaluate the performance of VL testing on DBS and DPS, in comparison to testing of plasma, using two common VL assays, the Roche Cobas Ampliprep/Cobas TaqMan CAP/CTM and the Abbott M 2000 real-time assays. The secondary objective was to further assess the limit of detection for DBS using the Abbott assay.

## Methods

### Study participant description

The participants were HIV-positive patients on ART seeking care and treatment management in clinical care facilities in Western, Kenya.

### Ethical approvals and informed consent

Ethical approval for the study was obtained from Kenya Medical Research Institute (KEMRI) Scientific Steering and Ethics Committees in Nairobi, Kenya and the U.S. Centers for Disease Control and Prevention (CDC) Institutional Review Board in Atlanta, Georgia. All study participants provided written informed consent for study participation.

### Specimen collection, preparation and storage

Specimens from ART-experienced patients who came for targeted VL monitoring were collected from clinical facilities in Western Kenya region from November 2012 to February 2013. Intravenous whole blood specimens were collected in ethylenediamine tetra acetic acid (EDTA) anticoagulant tubes. Plasma separation and spotting of DBS and DPS were done in the KEMRI/CDC HIV-Research clinical laboratory. Plasma was separated from whole blood specimens within 6 hours from the time of collection and 1 mL was aliquoted into sterile cryovials and stored at -70°C until testing was performed. For each whole blood and plasma specimen, two Whatman 903 cards were prepared, one for DBS and one for DPS, by spotting 75μL of whole blood and plasma respectively on each of the 5 spots, following manufacturers’ recommendations. The DBS and DPS were air dried at ambient temperature in laminar flow overnight and then packed with desiccant and humidity indicator cards. The DFS specimens were then shipped at ambient temperature to KEMRI/CDC HIV-Research testing laboratory. Within the four months of specimen collection, VL testing was done on plasma for routine patient monitoring, while DFS were stored at -30°C for later processing. Matched plasma, DBS, and DPS specimens were placed into four categories of 50 specimens each, based on the CAP/CTM plasma VL results in copies /mL (<400, ≥400-<10,000, ≥10,000-<100,000; and ≥100,000). Results <550 copies/mL were considered undetectable when using DBS and DPS for VL determination with Abbott M2000. While results <400 copies/mL were considered undetectable when using DBS and DPS for VL determination with the Roche CAP/CTM. Detectable plasma VL values for each assay platform were used as the reference group for comparison with the corresponding DBS and DPS VL values in assessing correlation and agreement. To assess the reproducibility of results from DBS and DPS VL testing using the Abbott test, 80 DBS and 80 DPS (40 with undetectable and 40 with detectable VL copies) selected randomly were re-processed in duplicates. To further assess the limit of detection (LOD) of the Abbott test, DBS panels obtained from CDC International Laboratory Branch were used. The DBS LOD panels were made from HIV-negative human blood spiked with commercially available HIV-1 panels with known VL concentrations (Acrometrix by Life Technology, Benicia, CA, USA). The spiked blood at 5,000, 2,500, 1,000, 500, and 250 copies /mL was then used to prepare DBS and processed in twenty replicates for each concentration to evaluate for the Abbott test LOD. Due to run failures there were limited number of spots to evaluate LOD for the CAP/CTM test.

### Nucleic acid processing and detection

#### Processing and detection with the Roche COBAS Ampliprep/COBAS Taqman

The CAP/CTM assay (Roche Diagnostics Ltd, Rotkruez, Switzerland) was used for automated extraction, amplification and quantification on plasma and DFS following the Roche manufacturer’s instructions. Briefly, 1000 μL of plasma specimens were aliquoted into the respective specimen tubes after a brief vortexing obtain a uniform mix. The specimens were transferred to the CAP/CTM for processing using the HI2CAP96 method[19]. For DFS the CAP/CTM HIV-1 dried fluid spot test procedure (HI2DFS96 version 2.0) was used. Extraction, amplification and quantitation for DFS were identical to those procedures for plasma specimens, with the following manufacturer’s modifications; one full DBS circle from each specimen was placed in a 1.8 mL specimen tube. To this tube, 1000 μL of specimen pre-extraction reagent was added for elution, the eluent was transferred to a thermomixer (Eppendorf AG, Hamburg, Germany) at 1000 rpm for 10 minutes at 56°C[19]. Thereafter, the specimens were transferred to the CAP/CTM for processing.

#### Processing and detection with the Abbott M2000

Plasma and DFS specimens testing using the Abbott m2000 sample preparation (sp)/Abbott m2000 real time (rt) analyzer (Promega, Madison, WI, USA) involved automated extraction, amplification and quantitation. Testing of plasma was done following manufacturer’s standard guidelines of 0.6 mL in the m2000 plasma protocol[20]. For DFS RNA extraction was done following the manufacturer’s procedure, briefly described as follows; for each specimen, two half-inch disks (two spots) were obtained from each card and placed in respectively labeled 15 mL propylene tube. Then, 1.7 mL of bulk m-lysis reagent, provided with the Abbott sample preparation assay, was added into each tube and incubated for 15 minutes, with intermittent mixing at room temperature, to lyse. The lysates were transferred to respective m2000sp reaction vessels. Processing was performed utilizing the 1mL DFS version 4.0 protocol on m2000sp, following manufacture’s guidelines[20]. Real time amplification and quantification was performed on m2000rt, also using the manufacture’s 1mL DBS/DPS protocol.

### Statistical analysis

HIV-1 RNA levels in both plasma and DBS specimens were transformed to log_10_ values. The values used had already been adjusted for hematocrit and sample volume using correction factors incorporated in both the CAP/CTM and Abbott assays software. Using the plasma VL for each assay as reference, the sensitivity, specificity, positive predictive value (PPV) and negative predictive value (NPV) (and 95% exact binomial Confidence Intervals [CI]) were calculated around the assay’s lower limit of detection (400cps/ml for CAP/CTM and 550cps/ml for Abbott). Concordance correlation and Bland Altman analyses were performed to determine precision and agreement of VL measurements between plasma and DBS and plasma and DPS specimens, with the CAP/CTM and Abbott assay, using plasma specimens as the reference group. To analyze reproducibility for the Abbott assay using DBS and DPS, the concordance correlation coefficient and Bland-Altman were utilized. We used probit analysis to determine the limit of detection of the Abbott assay using DBS, and the receiver-operating curve and likelihood ratios were used to estimate the various clinical relevant cut-offs for treatment failure. All statistical analyses were conducted using SAS, version 9.2 (SAS Inc., Cary, North Carolina, USA).

## Results

### Detection rates in DBS and DPS at corresponding VL levels

The proportion of DBS and DPS specimens with detectable VL increased with an increase in plasma VL level. Among the specimens with undetectable plasma VL, 96% with CAP/CTM and 14% with Abbott had detectable VL using DBS (Table 1). In addition, 6% with CAP/CTM and 2% with Abbott had detectable VL using DPS (Table 2).

**Table 1:** HIV-1 RNA detection rates of dried blood spot (DBS) specimens paired with plasma specimens prepared from patient support centers in Nyanza, Kenya. (November 2012 to February 2013).

	VL (log_10_ copies/mL) in corresponding paired plasma specimen	Number of paired DBS and plasma specimens tested	Number of DBS specimens with detectable VL	Proportion of total DBS specimens tested with detectable VL (%)
CAP/CTM	<2.6	50	48	96
	2.6–3.99	50	50	100
	4.0–4.99	50	50	100
	>5.0	50	50	100
	Total	200		
Abbott	<2.6	50	7	14
	2.6–3.99	50	41	82
	4.0–4.99	50	50	100
	>5.0	50	50	100
	Total	200		

**Table 2:** HIV-1 RNA detection rates of dried plasma spot (DPS) specimens paired with plasma specimens prepared from patient support centers in Nyanza, Kenya. (November 2012 to February 2013).

	VL (log_10_ copies/mL) in corresponding paired plasma specimen	Number of paired DPS and plasma specimens tested	Number of DPS specimens with detectable VL	Proportion of total DPS specimens tested with detectable VL (%)
CAP/CTM	<2.6	50	3	6
	2.6–3.99	50	42	84
	4.0–4.99	50	50	100
	>5.0	50	50	100
	Total	200		
Abbott	<2.6	50	1	2
	2.6–3.99	50	46	92
	4.0–4.99	50	50	100
	>5.0	50	50	100
	Total	200		

### Performance of DBS for VL determination using Roche and Abbott testing assays

**Fig 1:**
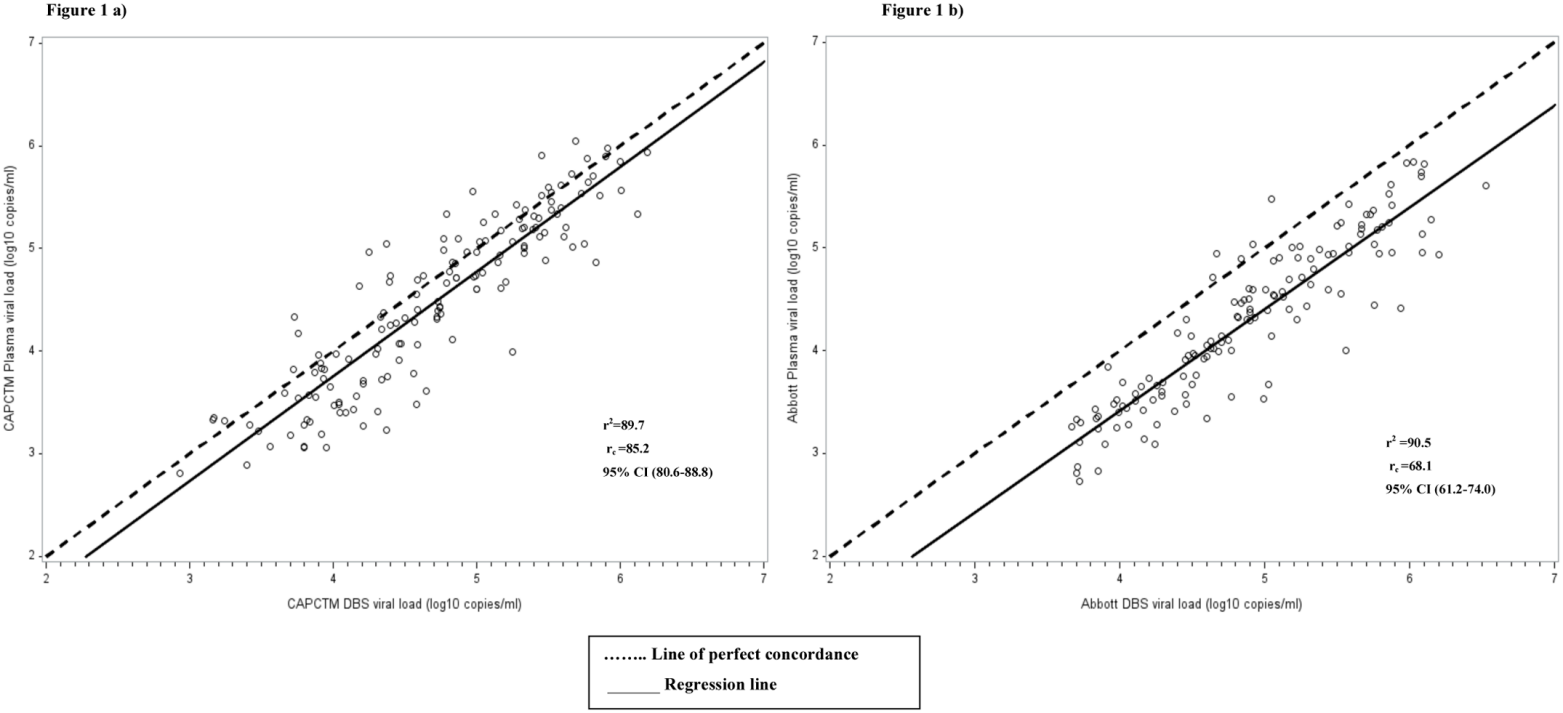
Concordance correlation analyses of HIV-1 (VL) quantification among plasma and DBS specimens collected from patients visiting Nyanza patient support centers and tested with CAP/CTM and Abbott. **a)** CAP/CTM plasma vs. DBS VL; **b**) Abbott plasma vs. DBS VL.

**Fig 2:**
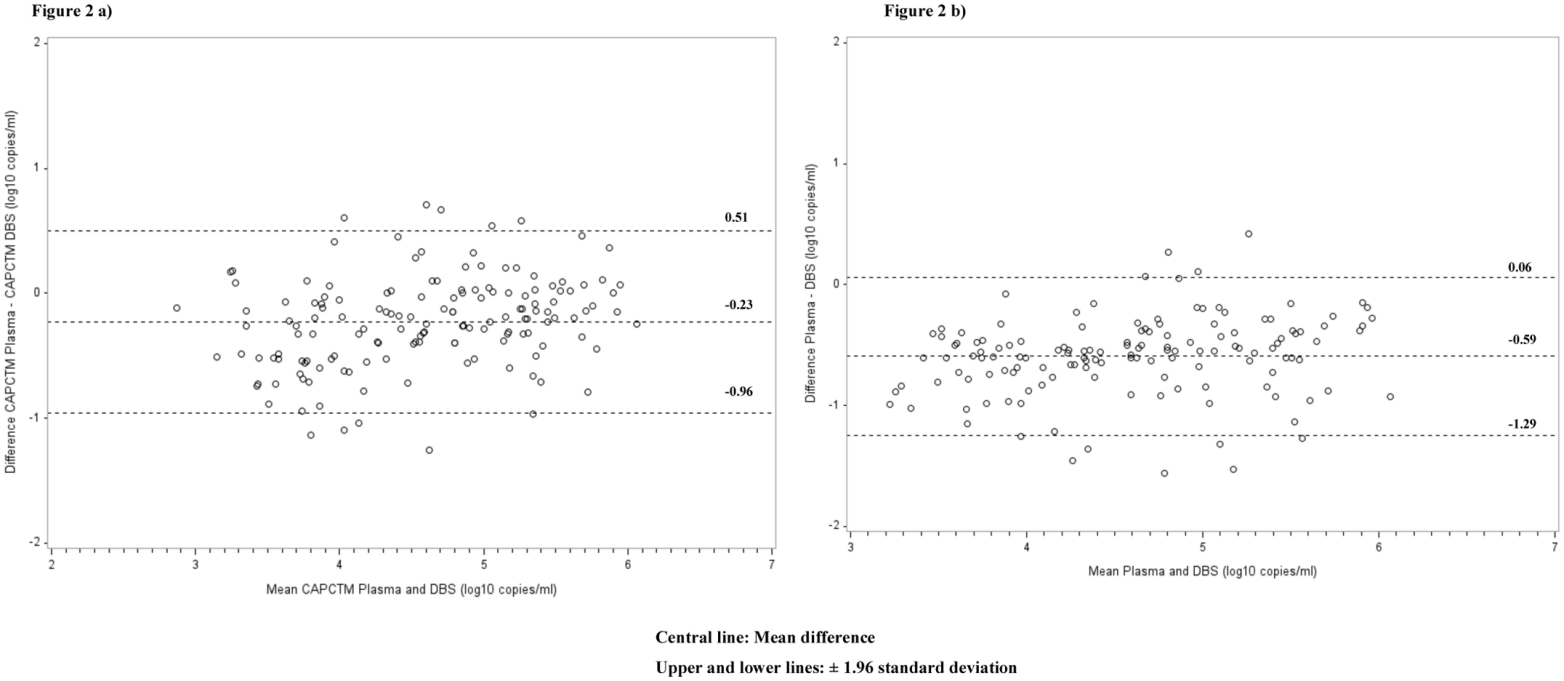
Bland-Altman analyses to evaluate agreement in HIV-1 (VL) quantification among plasma and dried blood spot specimens collected from patients visiting Nyanza patient support centers and tested with CAP/CTM and Abbott. The difference between the reference and the comparison specimen type were plotted against the average of the reference group and the comparison specimen type **a**) CAP/CTM DBS VL; **b**) Abbott DBS VL.

The sensitivity of VL detection for DBS using the CAP/CTM assay was significantly higher (100.0%, 95% CI: 97.6–100.0) compared to the Abbott assay (93.9%, 95% CI: 88.8–97.2). The specificity was however significantly higher for the Abbott assay (88.0%, 95% CI: 82.2–92.4) than CAP/CTM (4.0%, 95% CI: 0.5–13.7). The PPV for Abbott was also significantly higher (100.0%, 95% CI: 97.4–100.0) than CAP/CTM (75.8%, 95% CI: 69.2–81.6) but there was no much difference in the NPV for the two assays; CAP/CTM (100.0%, 95% CI: 15.8–100.0) and Abbott (85.5%, 95% CI: 73.8–93.0) (Table 3). For the CAP/CTM, the correlation between paired plasma and DBS VL values was strong, with a correlation coefficient of 89.7, P<0.001 and concordance correlation coefficient (r_c_) of 85.2, 95% CI: 80.6–88.8 (Fig 1a). Bland-Altman analysis showed mean difference (bias) of -0.23, and 95% limits of agreement of -0.96 to 0.51 log_10_ (Fig 2a), with 6% of the results (9/150) falling out of the **±** 1.96 standard deviation (SD). Using Abbott, there was a strong correlation between the paired plasma-DBS specimens VL values with a correlation coefficient of 90.5, P<0.001 and r_c_ 68.1 95% CI: 61.2–74.0 (Fig 1b). Bland-Altman analysis showed mean difference (bias) of -0.59 and 95% limits of agreement of -1.25 to 0.06 log_10_ (Fig 2b), with 6.7% (10/150) falling out of the **±** 1.96 standard deviation (SD).

**Table 3:** Sensitivity, specificity, positive predictive value (PPV), and negative predictive value (NPV) (95% confidence intervals) of dried blood spot (DBS) and dried plasma spot (DPS) compared with paired plasma specimen VL, patient support centers, Nyanza, Kenya. (November 2012 to February 2013).

		Plasma VL			
**CAP/CTM**	DBS VL	Detectable	Undetectable	Total	Sensitivity = 100.0 (97.6–100.0) Specificity = 4.0 (0.5–13.7) PPV = 75.8 (69.2–81.6) NPV = 100.0 (15.8–100.0)
	Detectable	150	48	198	
	Undetectable	0	2	2	
	Total	150	50	200	
**Abbott**	DBS VL	Detectable	Undetectable	Total	Sensitivity = 93.9 (88.8–97.2) Specificity = 88.0 (82.2–92.4) PPV = 100.0 (97.4–100.0) NPV = 85.3 (73.8–93.0)
	Detectable	137	0	137	
	Undetectable	11	52	63	
	Total	148	52	200	
**CAP/CTM**	DPS VL	Detectable	Undetectable	Total	Sensitivity = 94.7 (89.8–97.7) Specificity = 94.0 (83.5–98.8) PPV = 97.9 (94.1–99.6)
	Detectable	142	3	145	
	Undetectable	8	47	55	
	Total	150	50	200	
**Abbott**	DPS VL	Detectable	Undetectable	Total	Sensitivity = 97.3 (93.2–99.2) Specificity = 98.1 (89.7–100.0) PPV = 99.3 (96.2–100.0) NPV = 92.7 (82.4–98.0)
	Detectable	142	1	143	
	Undetectable	6	51	57	
	Total	148	52	200	

DBS viral load cut off of 400copies/mL and 550 copies/mL were used for CAP/CTM and Abbott respectively

### Performance of DPS for VL determination using Roche and Abbott testing assays

Performance measures for the Abbott and CAP/CTM tests were 97.3% (95% CI: 93.2–99.2) and 94.7% (89.8–99.7) for sensitivity, specificity 98.1% (89.7–100) vs. 94.0% (83.5–98.8) PPV 99.3% (96.2–100.0) vs. 97.9% (94.1–99.6), and NPV 92.7% (82.4–98.0) vs. 85.5% (73.3–93.5) respectively (Table 3).

**Fig 3:**
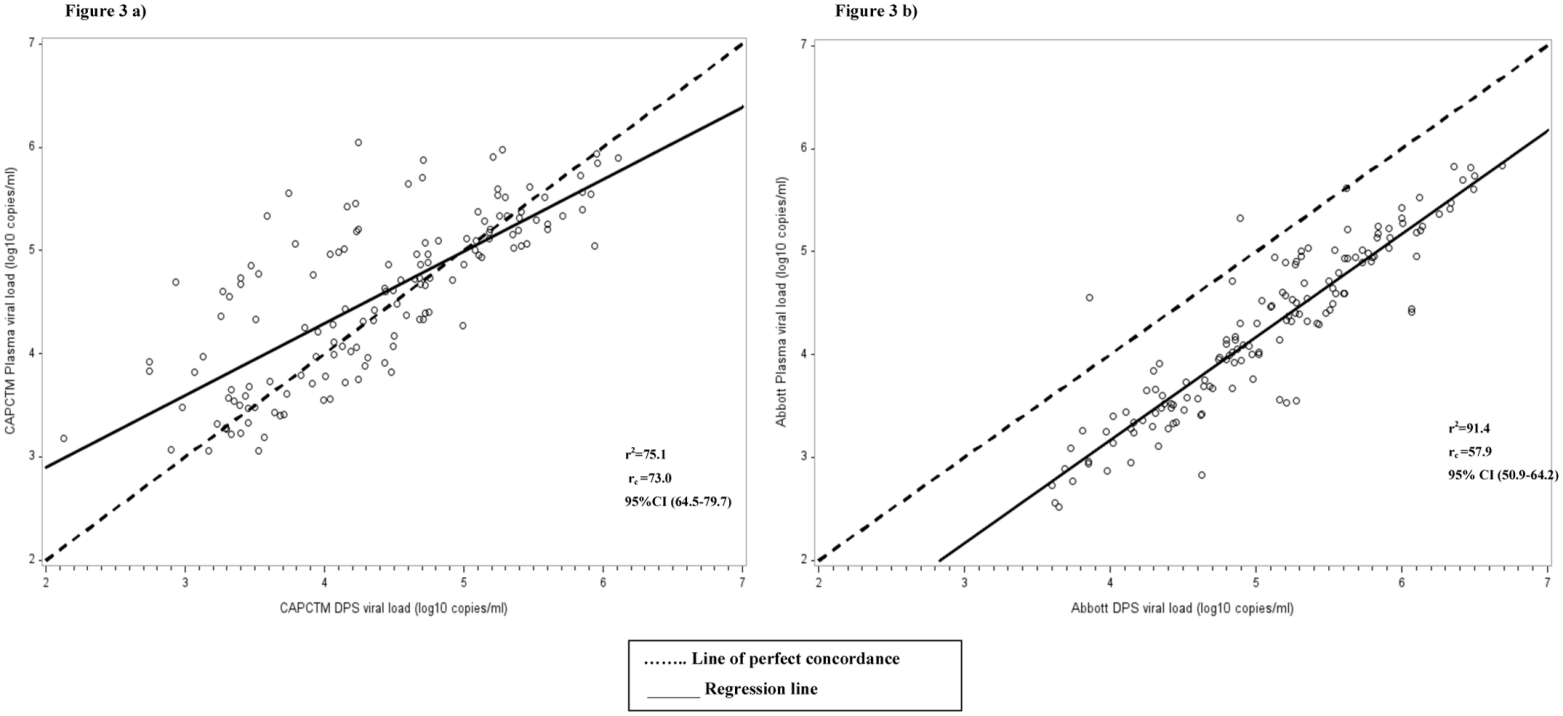
Concordance correlation analyses of HIV-1 viral load (VL) quantification among plasma and dried plasma spot specimens collected from patients visiting Nyanza patient support centers and tested with CAP/CTM and Abbott. **a)** CAP/CTM plasma vs. DPS VL; **b**) Abbott plasma vs. DPS VL.

**Fig 4:**
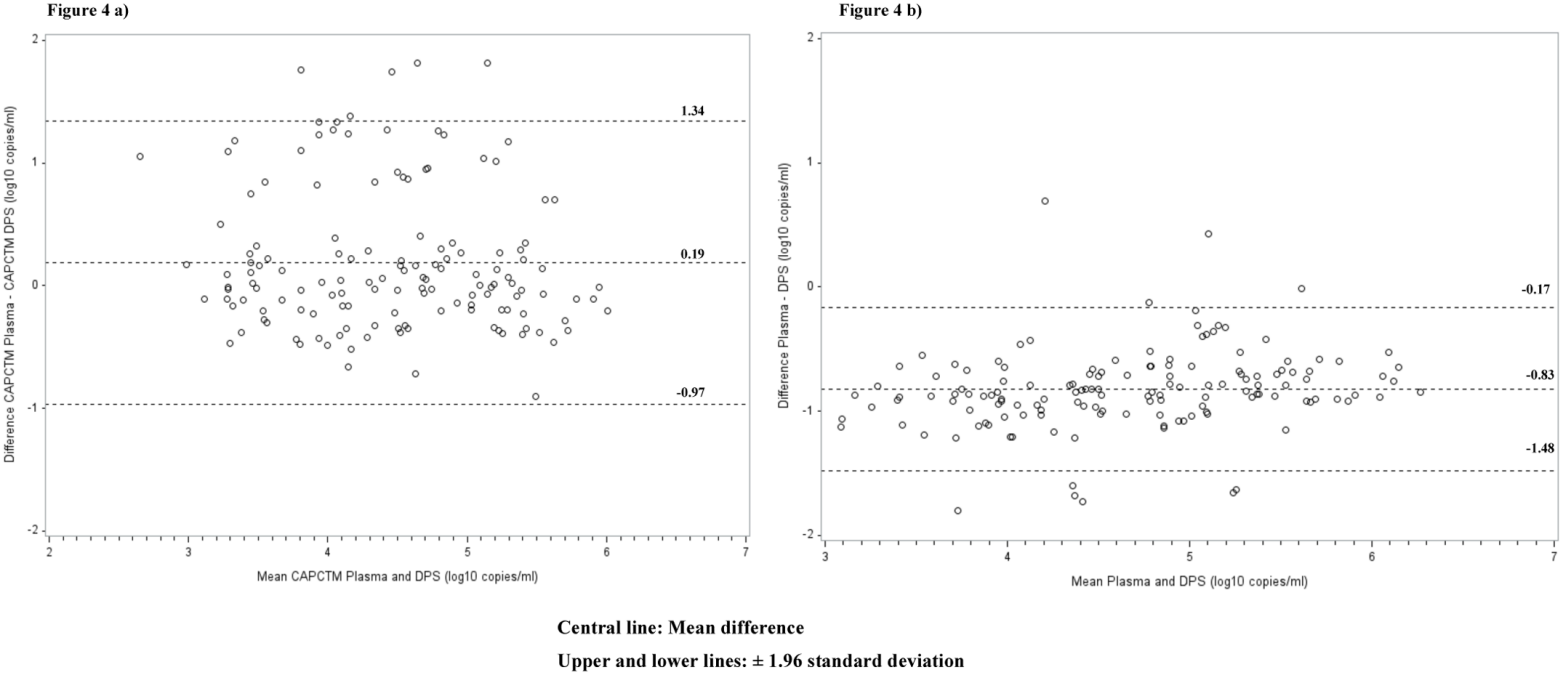
Bland-Altman analyses to evaluate agreement in HIV-1 viral load (VL) quantification among plasma and dried plasma spot specimens collected from patients visiting Nyanza patient support centers and tested with CAP/CTM and Abbott. The difference between the reference and the comparison specimen type were plotted against the average of the reference group and the comparison specimen type **a**) CAP/CTM DPS VL; **b**) Abbott DPS VL.

Strong correlation was observed between paired plasma and DPS VL values using the CAP/CTM assay, with a correlation coefficient (r^2^) of 75.1, P<0.001 and r_c_ of 73.0, 95% CI: 64.5–79.7 (Fig 3a). Bland-Altman analysis showed mean difference (bias) of 0.19 and 95% limits of agreement of -0.97 to 1.34 log_10_ (Fig 4a), with 3.3% (5/150) falling out of the **±** 1.96 standard deviation (SD). With the Abbott test, there was a strong correlation in VL results between the paired plasma and DPS specimens (r^2^ = 91.4, P<0.001 and r_c =_ 57.9, 95% CI: 50.9–64.2) (Fig 3b). Bland-Altman analysis showed mean difference (bias) of -0.83 and 95% limits of agreement of -1.48 to 0.17 log_10_ (Fig 4b), with 6.7% of the results (10/150) falling out of the **±** 1.96 standard deviation (SD).

Comparing paired plasma VL values with the CAP/CTM as the reference and Abbott as the comparison group, we observed a strong correlation (r^2^ = 91.8, P< 0.001 and rc = 87.1, 95% CI: 83.0–90.3). Bland-Altman analysis showed mean difference (bias) of -0.30 and 95% limits of agreement of -0.44 to 1.03 log_10_, with 4.7% (7/150) falling out of the **±** 1.96 standard deviation (SD).

### Reproducibility of DBS and DPS measurements on Abbott real-time assay

**Fig 5:**
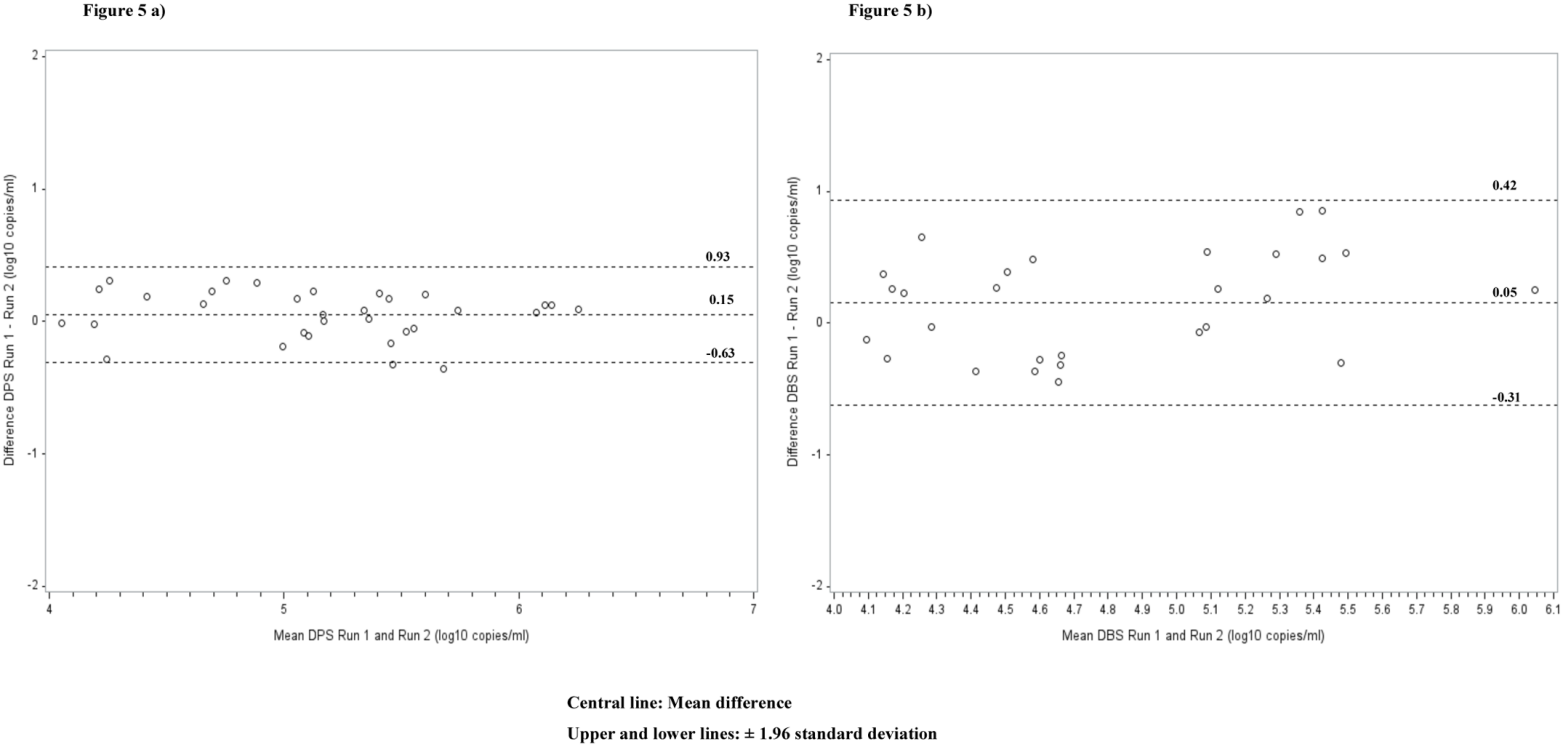
Bland-Altman analyses to evaluate repeatability in HIV-1 viral load quantification in 40 detectable a) DBS b) DPS collected from patients visiting Nyanza patient support centers and tested with Abbott. The difference between the references Run 1 and the comparisons Run 2 were plotted against the average of the reference group.

We further assessed the reproducibility of DBS and DPS on the Abbott system using 80 DBS and 80 DPS specimens. The results showed a strong r_c_ of 73.6, 95% CI: 51.6–86.5, with all the VL values falling within ± 2 standard deviations for DBS. For the 40 detectable DPS specimens, we observed a strong r_c_ of 95.3 (95% CI: 90.4–97.7). Bland-Altman analysis showed mean difference (bias) of 0.05 and 95% limits of agreement of -0.31 to 0.42 log_10_ for DPS and a bias of 0.15, 95% limits of agreement of -0.63 to 0.93 log_10_ for DBS (Fig 5).

### Limit of detection for DBS on Abbott system

**Fig 6:**
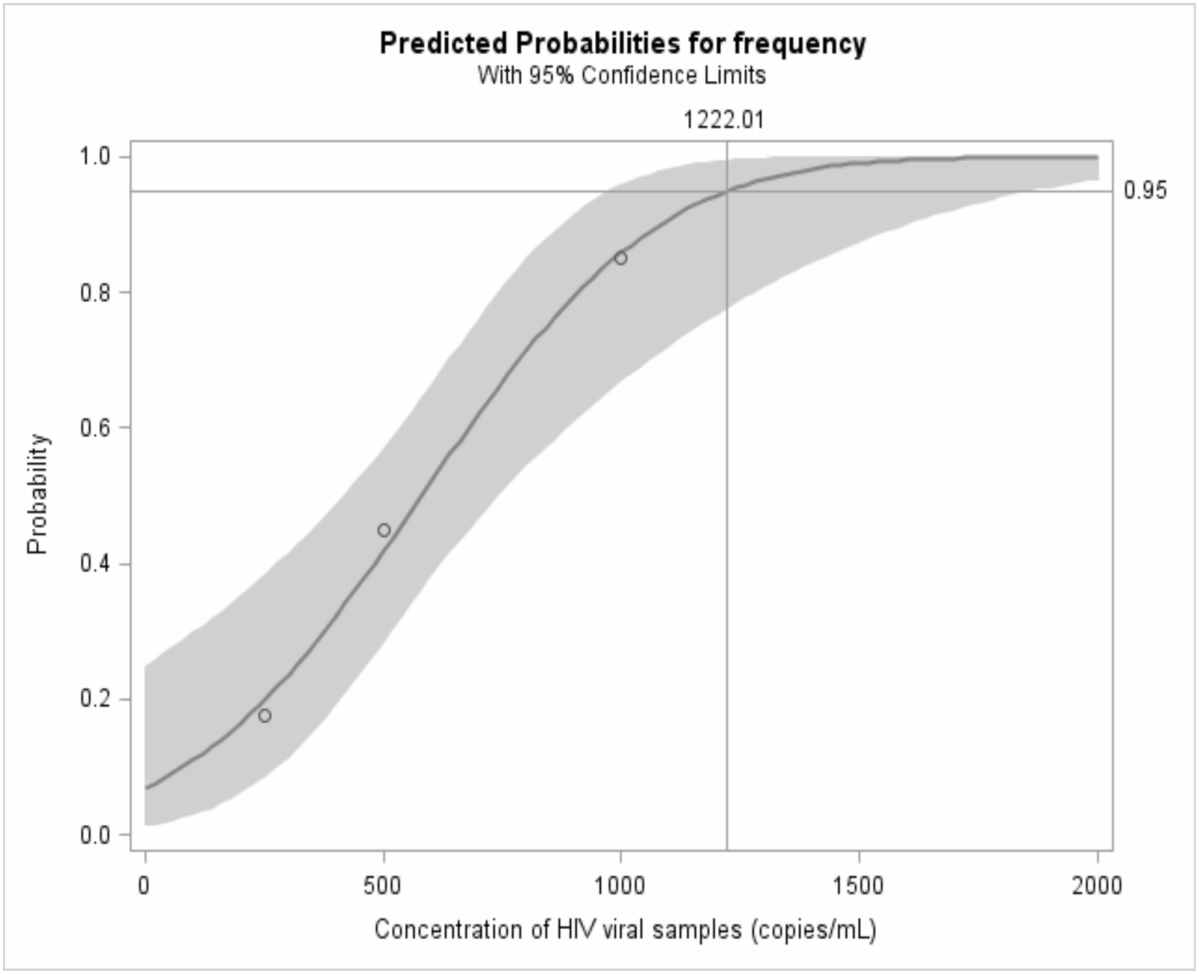
Probit analysis for Abbott dried blood spot viral load limit of detection. Limit of detection 1,222copies/mL, 95% CI (978.12, 1873.7).

Using probit analysis for the DBS panel replicates, a limit of detection of 1,222 copies/mL (95% CI: 978, 1874) was observed (Fig 6). The assay performance in the selected VL categories was 100% at 2500 and 5000 copies/mL, 85% at 1000 copies/mL, 45% at 500 copies/mL, and 17.6% at 250 copies/mL.

### Determination of optimal clinical threshold for VL failure determination using DBS

Further assessment to determine the DBS VL threshold in which the assays had optimum performance, when using CAP/CTM plasma as reference, showed the best cut-off for CAP/CTM at 4000 copies /mL, with 92.5% accuracy (sensitivity of 96.0% [95%CI 91.0–98.3] and specificity of 82.7% [95%CI 69.2–91.3]). Likewise, the best threshold for the Abbott real-time assay was 3,000 copies /mL with 95.5% accuracy (sensitivity 94.6% [95%CI 89.3–97.5], specificity 98.1% [95%CI 88.4–99.9]) (Table 4). This however, did not differ substantially from 1000 copies/ml with 95% accurate detections (sensitivity 96.6% [95% CI 91.8–98.7], specificity 90.4% [95% CI 78.2–96.4]), nor any of the other Abbott System cutoffs (positive Likelihood Ratio [LR+] >10%) at any cutoff as compared to CAP/CTM (LR+ with <3% at ≤3000). The AUC of the ROC for DBS VL using CAP/CTM and Abbott, respectively, was 0.90 (95% CI: 0.85–0.96) and 0.97 (95% CI: 0.95–1.0).

**Table 4:** Comparisons between CAP/CTM dried blood spot and Abbott dried blood spot tests at different cut-offs using CAP/CTM plasma as the gold standard.

Cut-point (Viral copies/mL)	Test	Sensitivity (%)	Specificity (%)	Correctly classified (%)	LR+*	LR-**
**≥1000**	CAP/CTM	100	17.3	78.5	1.21	0.00
**≥1000**	Abbott	96.6	90.4	95.0	10.0	0.04
**≥2000**	CAP/CTM	98.0	36.5	82.0	1.54	0.06
**≥2000**	Abbott	95.3	94.2	95.0	16.5	0.05
**≥3000**	CAP/CTM	97.3	54.0	86.0	2.11	0.05
**≥3000**	Abbott	94.6	98.1	95.5	49.2	0.06
**≥4000**	CAP/CTM	96.0	82.7	92.5	5.54	0.05
**≥4000**	Abbott	93.2	98.1	94.5	48.5	0.07
**≥5000**	CAP/CTM	95.3	84.6	92.5	6.19	0.06
**≥5000**	Abbott	93.0	98.1	94.0	48.1	0.07

*LR+: Positive likelihood ratio

**LR- Negative likelihood ratio

## Discussion

The logistical constraints presented by plasma as the specimen type for VL testing in resource limited settings may create restricted access to VL testing. Following the recent WHO guidelines, countries in sub-Saharan Africa have adopted routine VL monitoring. However, implementation is hampered by, among others, suitable specimen type for patients from remote areas. The performance of DFS is an alternative that could overcome the logistical constraints associated with plasma collection and shipment need to be assessed. The current study evaluated the performance of DBS and DPS for VL testing using CAP/CTM and Abbott assays.

Both CAP/CTM and Abbott showed good sensitivity for DBS VL, which is supported by results from similar studies[14,21,22] but the specificity was comparatively higher for the Abbott assay. The use of DBS for VL-tests has generally been reported to result with low specificity especially for the CAP/CTM assay[23–26]. A possible explanation could be the contribution of HIV-1 pro-viral DNA and viral-RNA from peripheral blood mononuclear cells in RT-PCR[27]. The observed difference in specificity between Abbott and CAP/CTM assays could be attributed to the ability of the former to exclude the contamination from pro-viral DNA. CAP/CTM extraction technology using silica beads does not discriminate between HIV-1 DNA and RNA[19], while the Abbott assay discriminates against HIV-1 intracellular DNA, extracting mainly the RNA, using iron oxide beads[20]. Hypothetically, the contribution of HIV cell-associated DNA and RNA may be the source of false detectable VL in DBS in samples with undetectable plasma VL[11,28]. This could further be the reason for the slightly lower specificity for the Abbott assay as it further fails to discriminate cellular RNA from cell-free RNA. The NucliSense nucleic acid sequence based amplification (NASBA), which specifically amplifies single stranded RNA by use of T7 RNA polymerase[29] has been shown to result with a higher specificity than the Abbot assay[30]. These findings continue to highlight the need for assay-specific recommendations to guide DBS VL testing. Alternatively, more studies should be carried out to optimize the use of DBS in viral load tests especially those targeting extraction methods that discriminate cell-free RNA from cell-associated RNA or DNA. Limited studies assessing on such methods show promise, including the free viral elution protocol by Roche[31] and the leucocyte depletion method incorporated in the whole-blood based SAMBA point-of-care test[32].

The performance of DPS was comparable to plasma, supporting its utility as an alternative specimen type to plasma for VL testing. However, due to requirements of centrifugation to separate plasma, and cold chain processing to maintain specimen integrity, use of DPS may not serve the purpose of increasing access to VL testing in RLS. Despite this limitation, technologies have been developed to minimize the complexities associated with DPS collection, including low cost, rapid, and simple to use plasma separators that require low volume of whole blood[33], which could provide an opportunity to expand utilization of DPS for VL testing. DBS and DPS gave reproducible results using the Abbott platform both at low and high VL levels. This finding is consistent with findings from other studies[18,22]. WHO guidelines recommend a cut-off of 1,000 copies /mL for treatment failure for both plasma and DBS specimens[3]. We however observed a trade-off in specificity and sensitivity whilst using the current WHO threshold for DBS (1000cps/ml) and the 2013 recommended threshold of 3000–5000 cps/ml. At 1,000 copies /mL nearly 10% of patients would be misclassified by the Abbott assay as falsely failing treatment and about 4% as falsely responding to ART. The CAP/CTM assay with a perfect sensitivity but a much lower specificity would lead to nearly 83% of patients being falsely misclassified as failing treatment. Increasing the cut-off improves the specificity of the CAP/CTM to a maximum best of 83% at 4,000 copies /mL. For the Abbott assay, the specificity increases to its maximum best at 3000cps/ml, reducing the false misclassification rate for treatment failure from 10% to 2%. However the risk of missing patients failing treatment increases from 3.4 to 6.4%. Similar findings have been observed in other studies and suggests the need for a trade-off in the use of DBS-based assays[26,34]. Lowering the cut-off minimizes contamination from cellular viral nucleic acid, but in turns increases the risk of missing patients failing treatment with low-levels of viremia[26,34]. However, it is important to note that clinical decisions are dependent on an algorithm that requires a confirmatory test 3 months after intensive adherence counseling. In such an algorithm, a lower cut-off for virological failure (1000cps/ml) could result with increased costs for repeat testing but more persons failing treatment could be detected much early. On the other hand a higher cut-off such as 3000cps/ml could reduce the costs for repeat testing when using the Abbott assay but potentially miss 3% of cases failing treatment. As it is unclear how these tests would perform within the current algorithm, further studies are needed to determine the best assay performance characteristics that would maximize the use of DBS.

To our knowledge this is the first study in Kenya to evaluate and compare the use of DBS and DPS to plasma specimens for VL testing using both Abbott and CAP/CTM assays. Since both assays are commonly used in Kenya, the findings of this study will provide guidance to the Kenya Ministry of Health in implementing use of DBS for VL testing to meet the increased demand of routine VL testing. However, further studies are needed to evaluate the performance of VL testing of DBS collected, shipped and stored at ambient temperatures in field conditions. One such study has been carried out in Kenya, evaluating not only venous DBS, but finger stick and micro capillary DBS for VL testing, with comparable results to plasma[13] (Schmitz, 2014), This study had some limitations. First, due to a limited number of DBS and DPS specimens, analysis on LOD was done only on the Abbott assay; additionally, specimens were from ART-experienced patients with missing information on treatment history. Future studies should assess whether duration of ART use could influence the performance of DBS for VL testing.

In conclusion, good correlation and agreement were observed between matched DBS, DPS and plasma for Abbott, while good correlation was observed for DPS and plasma using CAP/CTM. Our findings show that DBS and DPS may be reliably used as alternative specimens to plasma to measure HIV-1 VL using Abbott, while DPS may be reliably used with CAP/CTM in resource-limited settings.
